# Amphetamine Modulation of Long-Term Object Recognition Memory in Rats: Influence of Stress

**DOI:** 10.3389/fphar.2021.644521

**Published:** 2021-02-24

**Authors:** Paola Colucci, Alessia Santori, Luca Romanelli, Clemens Zwergel, Antonello Mai, Sergio Scaccianoce, Patrizia Campolongo

**Affiliations:** ^1^ Dept. of Physiology and Pharmacology, Sapienza University of Rome, Rome, Italy; ^2^ Neurobiology of Behavior Laboratory, Section of Neuropsychopharmacology, IRCCS Santa Lucia Foundation, Rome, Italy; ^3^ Dept. of Drug Chemistry & Technologies, Sapienza University of Rome, Rome, Italy

**Keywords:** memory consolidation, forced swim stress, norepinephrine, adrenal medullectomy, posttraumatic stress disorder

## Abstract

Amphetamine is a potent psychostimulant that increases brain monoamine levels. Extensive evidence demonstrated that norepinephrine is crucially involved in the regulation of memory consolidation for stressful experiences. Here, we investigated amphetamine effects on the consolidation of long-term recognition memory in rats exposed to different intensities of forced swim stress immediately after training. Furthermore, we evaluated whether such effects are dependent on the activation of the peripheral adrenergic system. To this aim, male adult Sprague Dawley rats were subjected to an object recognition task and intraperitoneally administered soon after training with amphetamine (0.5 or 1 mg/kg), or its corresponding vehicle. Rats were thereafter exposed to a mild (1 min, 25 ± 1°C) or strong (5 min, 19 ± 1°C) forced swim stress procedure. Recognition memory retention was assessed 24-h after training. Our findings showed that amphetamine enhances the consolidation of memory in rats subjected to mild stress condition, while it impairs long-term memory performance in rats exposed to strong stress. These dichotomic effects is dependent on stress-induced activation of the peripheral adrenergic response.

## Introduction

The psychostimulant amphetamine was discovered more than a century ago (see Heal et al., 2013 for a review). Chemical structure analogies among amphetamine and other monoamine neurotransmitters, such as norepinephrine, dopamine and serotonin, are crucial not only for the amphetamine’s mechanism of action, but also for its pharmacological properties (Ferris and Tang, 1979). It is well known that amphetamine acts as a competitive substrate of the norepinephrine, dopamine and serotonin re-uptake transporters (NET, DAT and SERT, respectively) (Sulzer et al., 2005). Once entered in the presynaptic neuron, amphetamine disrupts the monoamine storage vesicles and, consequently, increases monoamine levels in the neural cytosolic pool (Teng et al., 1998). Such enhanced cytosolic concentration of monoamines reverts the transport direction of NET, DAT and SERT, thus increasing the amount of norepinephrine, dopamine and serotonin in the synaptic cleft (Robertson et al., 2009). It has been demonstrated that augmented levels of monoamines, in particular norepinephrine and dopamine, at the synaptic terminal, are responsible for euphoria, mood improvements and the general sense of wellbeing induced by amphetamine intakes (De Wit et al., 2002; Pester et al., 2018). Literature data demonstrated that amphetamine induces profound effects on learning and memory processes. While it generally enhances memory consolidation, it has been shown that it increases memory retrieval errors and alters working memory performances (Martinez et al., 1980a; Ballard et al., 2014; Bardgett et al., 2019). Interestingly, it has been shown that amphetamine effects on memory consolidation are dependent on the amphetamine-induced activation of the noradrenergic system (Lee and Ma, 1995; Colucci et al., 2019), a neurotransmitter system critically involved in the modulation of long-term memory consolidation (Ferry et al., 1999; Roozendaal and McGaugh, 2011; McLumiere et al., 2017). We recently demonstrated that the dissociative drug ketamine enhances memory performance through a mechanism that activates both the central and peripheral noradrenergic signaling (Morena et al., 2017; Morena et al., 2020).

It is widely recognized that emotionally arousing experiences, which activate the endogenous stress systems, are well remembered over time (McGaugh, 2006). The activation of the hypothalamic–pituitary–adrenal (HPA) axis, mediated by the stress response, culminates with the release, by the adrenal glands, of stress hormones: epinephrine, from the adrenal medulla, and glucocorticoids, from the adrenal cortex (Biddie et al., 2012). Such stress hormones finely tune the noradrenergic tone in the central nervous system (Smith and Vale, 2006) and modulate cognitive function, with an inverted U-shaped dose-effect curve (Schilling et al., 2013), by which optimal levels of stress hormones are responsible for memory potentiation, whereas their maladaptive expression leads to memory impairment (Salehi et al., 2010; McEwen, 2013).

Hence, considering the amphetamine modulation of noradrenergic system, which in turn influences memory processes, and taking into account that different stress intensities distinctly prompt stress hormone levels with divergent effects on cognitive functions, here we first aimed at investigating amphetamine effects on the consolidation of long-term recognition memory in rats that were exposed to different levels of stress. In a second set of experiments, we further evaluated whether the effects of amphetamine on long-term recognition memory consolidation were dependent on the activation of the peripheral adrenergic system, soon after exposure to different stress conditions.

## Materials and Methods

### Animal Care and Use

Male adult Sprague-Dawley rats (12 weeks old and 350–450 g at the time of training and testing, Charles River Laboratories, Calco, Italy) were kept individually in an air-conditioned colony room (temperature: 21 ± 1°C; lights on from 07:00 AM to 7:00 PM) with pellet food and water available ad libitum. Training and testing were performed during the light trial of the cycle between 11:00 AM and 2:00 PM. All procedures involving animal care or treatments were performed in compliance with the ARRIVE guidelines, the Directive 2010/63/EU of the European Parliament, and the D.L. 26/2014 of the Italian Ministry of Health.

### Drug Treatment

Amphetamine ((RS)-1-phenylpropan-2-amine) (0.5 and 1 mg/kg) was dissolved in saline 0.9% (vehicle) and administered intraperitoneally (i.p.) in a volume of 1 ml/kg, immediately after the training trial. Doses were chosen on the basis of pilot experiments performed in our laboratory and on literature data (Roozendaal et al., 1996; Colucci et al., 2019). The solutions were freshly prepared on the day of the experiment and protected from exposure to light.

### Behavioral Procedures

Object recognition task. A previously validated object recognition (OR) procedure described by Campolongo et al. (2013) was used. The experimental apparatus consisted of a grey open-field box (in cm, 40 wide × 40 deep × 40 high) with the floor covered with sawdust, positioned in a dimly illuminated room. The objects to be discriminated were transparent glass vials (5.5 cm diameter and 5 cm height) and white glass light bulbs (6 cm diameter and 11 cm length). All rats were handled twice per day for 1 min each and extensively habituated to the experimental context twice per day for 3 min each for 7 days preceding the training day. During habituation, rats were allowed to explore the apparatus in the absence of objects freely. The animals were randomly assigned to two different groups: mild and strong stress conditions. On the training trial, each rat was individually placed in the experimental apparatus at the opposite end from the objects. Rats were allowed to explore two identical objects (A1 and A2) for 6 min, then they were removed from the apparatus and, after drug treatment, according to the stress condition group, were subjected to a mild or strong swim stress procedure; subsequently, each rat was returned to the home cage. To avoid the presence of olfactory trails, sawdust was stirred, fecal boli were removed and the objects were cleaned with 70% ethanol after each trial. Rat’s behavior was recorded by a video camera positioned above the experimental apparatus and videos were analyzed with Observer XT 12 (Noldus Information Technology BV, Wageningen, The Netherlands) by a trained observer who was unaware of treatment condition. Exploration of an object was defined as pointing the nose to the object at a distance of <1 cm and/or touching it with the nose. Turning around or sitting on an object was not considered as exploration. During the training trial, the time spent exploring the two objects (total object exploration time, s) was taken as a measure of object exploration, and the exploratory behavior of the experimental apparatus was analyzed by measuring the total number of crossings and rearings. For crossings, the floor of the apparatus was divided into four imaginary squares and the total number of crossings between squares was determined. Long-term memory retention was tested 24-h after the training trial. On the testing trial, one copy of the familiar object (A3) and a new object (B) were placed in the same location as stimuli during the training trial. All combinations and locations of objects were used to reduce potential biases due to preference for particular locations or objects. Each rat was placed in the apparatus for 6 min, and its behavior was recorded. To analyze cognitive performance, during the retention test the preference of rats for objects was evaluated and a discrimination index (DI) was therefore calculated as the difference in time exploring the novel (B) and the familiar object (A3), expressed as the percentage ratio of the total time spent exploring both objects (B + A3).

Forced swim stress procedure. This procedure was carried out accordingly to Santori and colleagues (Santori et al., 2019; Santori et al., 2020). Immediately after the training trial of the OR task rats were forced to swim in a tank (50 cm in height × 20 cm in diameter), filled to a depth of 30 cm with water, in a separate room from that where the OR task was performed. Thereafter, rats were removed from the water and carefully wiped to dryness with absorbent paper before returning to the home cage. Mild and strong stress condition rat groups were subjected to a 1- or 5-min forced swim stress procedure at different water temperatures of 25 ± 1°C or 19 ± 1°C, respectively, known to elicit different plasma corticosterone levels (Morena et al., 2015; Santori et al., 2019).

### Surgical Procedures

Adrenal medullectomy. In a second set of experiments, rats were subjected to adrenal medullectomy, which was performed as previously reported in literature (Martinez et al., 1980b; Wilkinson et al., 1981; Shin et al., 2017). Summarily, each rat was anesthetized with a mixture of Zoletil and Domitor (40 mg/kg and 35 µg/kg respectively), given i. p. Animals were placed on a flat surface with their limbs in the extended position and their dorsal area was trichotomized. An incision of 2 cm was made on the right and left dorsal lateral surface of the animal just over each kidney. The overlying adipose tissue was removed, and it was possible to identify the adrenal glands. Small incisions were made on the adrenal capsule and the medulla was gently squeezed out. The wound was closed with an autoclip. Sham surgery was performed in the same manner, except for the removal of adrenal medullae. Consistently with literature data, rats were provided with drinkable 0.45% saline and allowed to recover from surgery for at least 7 days before experimental procedures (Khasar et al., 2009).

### Data and Statistical Analysis

One-sample t-tests were used to determine whether the DI was different from zero. OR data were analyzed by two-way ANOVA. When appropriate, Tukey-Kramer post hoc tests were used to determine the source of the detected significances. *p* values of <0.05 were considered statistically significant. To be included in the statistical analysis rats had to reach a minimum criterion of total object exploration time >10 s on either training or testing. Prior findings indicate that such rats adequately acquire the task (Okuda et al., 2004; Campolongo et al., 2013). All data are expressed as mean ± standard error of the mean (SEM) and each group’s n is indicated in the corresponding figure legend.

## Results

### Amphetamine Enhances Long-Term Memory Consolidation in Rats Subjected to the Mild Stress Condition While Impairs it in Rats Subjected to the Strong Stress Condition

This experiment investigated whether amphetamine administration, immediately after the training trial, modulates long-term memory consolidation in an OR task, when animals were subjected to a mild or strong forced swim stress condition.

Training trial. Two-way ANOVA for total exploration time of the two identical objects on the training trial, before drug administration and stress exposure, revealed no significant effect of post-training treatment (F_(2,51)_ = 1.048, *p* = 0.358), of post-training stress condition (F_(1,51)_ = 0.450, *p* = 0.505) and of the interaction between the two factors (F_(2,51)_ = 0.267, *p* = 0.767, Table 1). Examination of rats’ exploratory behavior of the experimental apparatus during the training trial indicated that there were no significant differences among groups for the number of crossings or rearings before drug treatment and stress exposure (Table 1). In fact, two-way ANOVA for the number of crossings or rearings on the training trial revealed no significant post-training treatment effects (F_(2,51)_ = 1.489, *p* = 0.235 and F_(2,51)_ = 1.602, *p* = 0.212, respectively), no significant post-training stress condition effects (F_(1,51)_ = 2.817, *p* = 0.099 and F_(1,51)_ = 0.143, *p* = 0.707, respectively) and no significant effect for the interaction between the two factors (F_(2,51)_ = 0.090, *p* = 0.914 and F_(2,51)_ = 0.516, *p* = 0.600, respectively).

**TABLE 1:** Exploratory behavior on the training trial for post-training vehicle- and amphetamine-treated rats that were subjected to mild or strong stress conditions immediately after training.

	Total object exploration time (s)	Number of crossings	Number of rearings
Mild stress			
Vehicle	76.8 ± 5.7	32.7 ± 2.8	42.9 ± 4.7
Amphetamine 0.5	80.4 ± 13.2	30.0 ± 3.1	38.1 ± 3.7
Amphetamine 1	63.6 ± 5.9	27.4 ± 3.8	63.6 ± 5.9
Strong stress			
Vehicle	79.5 ± 6.5	38.2 ± 3.8	44.6 ± 3.5
Amphetamine 0.5	79.9 ± 7.1	32.9 ± 2.4	38.4 ± 1.3
Amphetamine 1	74.6 ± 8.8	32.5 ± 3.5	35.2 ± 3.9
Sham			
Mild Stress			
Vehicle	72.5 ± 4.9	33.3 ± 3.1	40.6 ± 5.8
Amphetamine 1	61.1 ± 6.8	29.9 ± 4.0	40.1 ± 3.7
Strong Stress			
Vehicle	73.2 ± 6.8	38.5 ± 3.6	42.8 ± 1.3
Amphetamine 1	83.5 ± 9.7	33.3 ± 3.1	35.7 ± 3.7
Medullectomy			
Mild stress			
Vehicle	82.5 ± 8.5	28.8 ± 5.0	39.5 ± 2.5
Amphetamine 1	88.2 ± 7.6	30.3 ± 3.4	37.2 ± 2.9
Strong stress			
Vehicle	69.7 ± 5.6	26.9 ± 2.9	35.7 ± 3.9
Amphetamine 1	82.8 ± 7.7	26.6 ± 3.1	37.7 ± 4.3

**FIGURE 1:**
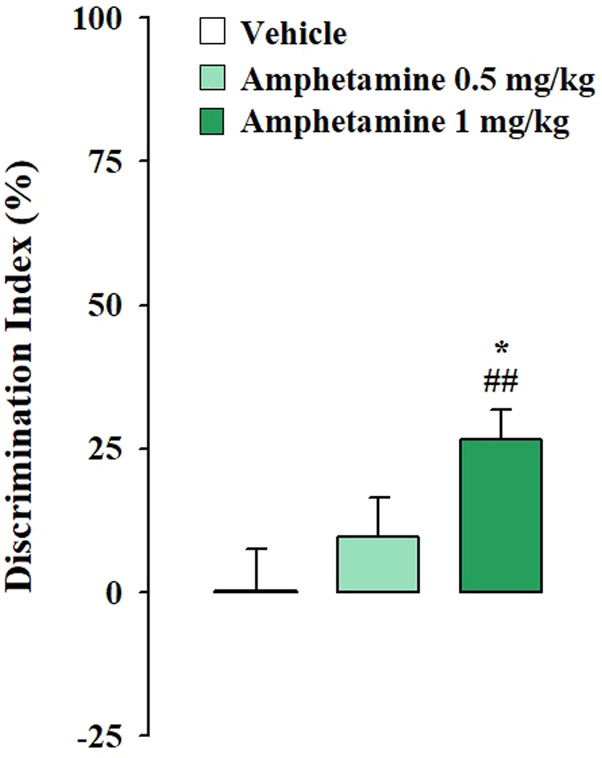
Amphetamine effects on the consolidation of long-term OR memory in rats exposed to the mild stress condition immediately after training. DI on the testing trial for vehicle- and amphetamine-treated rats that were subjected to the mild stress condition immediately after training. *Post hoc* comparisons reported significant differences between groups as follows: *p < 0.05 vs the corresponding vehicle group. ##*p* < 0.01, one-sample t-test significantly different from zero. Data are expressed as mean ± SEM (n = 8–9 per group).

**FIGURE 2:**
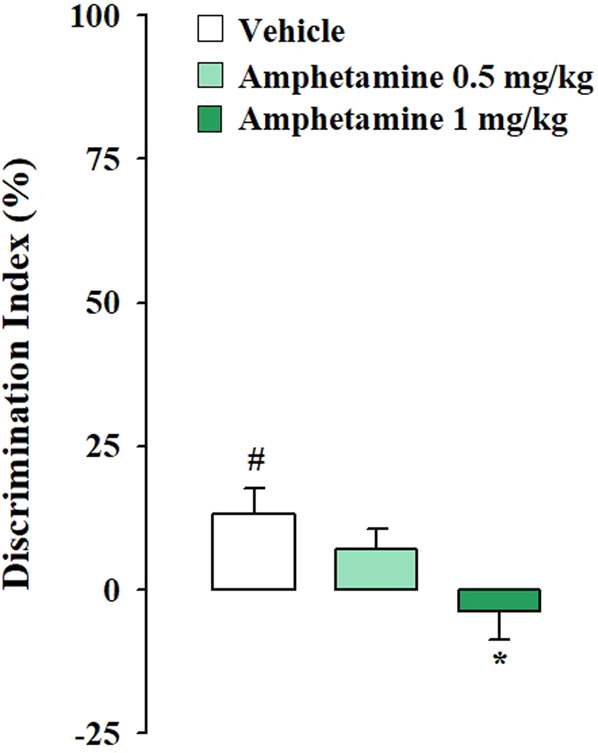
Amphetamine effects on the consolidation of long term OR memory in rats exposed to the strong stress condition immediately after training. DI on the testing trial for vehicle- and amphetamine-treated rats that were subjected to the strong stress condition immediately after training. *Post hoc* comparisons reported significant differences between groups as follows: *p < 0.05 vs the corresponding vehicle group. #*p* < 0.05, one-sample t-test significantly different from zero. Data are expressed as mean ± SEM (n = 10–11 per group).

Testing trial. As expected, according to the norepinephrine dose-response U-shaped curve on memory performance (Baldi and Bucherelli, 2005), vehicle-treated rats subjected to the mild stress condition did not express long-term memory retention for the familiar object, in fact the one sample t-test analysis revealed that their DIs were not significantly different form zero (t_(8)_ = 0.028, *p* = 0.978). Conversely, vehicle-treated rats subjected to strong stress condition discriminated the novel object with respect to the familiar one, as indicated by the one sample t-test analysis for their DIs (t_(9)_ = 3.007, *p* = 0.015). Rats that were administered with amphetamine at the dose of 0.5 mg/kg did not discriminate the two objects neither in the mild stress condition nor in the strong one (t_(7)_ = 1.378, *p* = 0.211: t_(10)_ = 1.930, *p* = 0.082, respectively). However amphetamine treatment at the dose of 1 mg/kg allowed rats to discriminate the two objects only when exposed to the mild stress condition (t_(8)_ = 5.078, *p* = 0.010) but not when exposed to the strong one (t_(9)_ = −0.765, *p* = 0.464). Two-way ANOVA analysis for the DI reported no significant treatment or stress effect (F_(2,51)_ = 0.391, *p* = 0.678; F_(1,51)_ = 2.254, *p* = 0.139, respectively) but a significant effect of the interaction between the two factors was detected (F_(2,51)_ = 8.423, *p* = 0.001). Post hoc analysis indicated that the DI of rats treated with 1 mg/kg of amphetamine and subjected to the mild stress condition was significantly higher with respect to that of the respective vehicle-treated rats (*p* < 0.05) (Figure 1). On the contrary, the DI of rats treated with 1 mg/kg of amphetamine and subject to the strong stress condition was significantly lower than that of the relative vehicle-treated rats (*p* < 0.05) (Figure 2). Two-way ANOVA for the total object exploration time on the testing trial indicated no significant effect of the treatment (F_(2,51)_ = 0.909, *p* = 0.409), a tendency toward significance for the stress effect (F_(1,51)_ = 3.959, *p* = 0.052) and no significant effect for the interaction between the two factors (F_(2,51)_ = 0.567, *p* = 0.571). Two-way ANOVA for the number of crossings revealed no significant effect of the treatment (F_(2,51)_ = 1.344, *p* = 0.270), a significant effect of the stress condition (F_(1,51)_ = 7.605, *p* = 0.008) and no significant effect for the interaction between the two factors (F_(2,51)_ = 1.355, *p* = 0.267). Finally, two-way ANOVA for the number of rearings indicated no significant effect for the treatment (F_(2,51)_ = 1.413, *p* = 0.253), for the stress condition (F_(1,51)_ = 0.361, *p* = 0.551) and for the interaction between the two factors (F_(2,51)_ = 0.231, *p* = 0.795) (Table 2).

**TABLE 2:** Exploratory behavior on the testing trial for vehicle- and amphetamine-treated rats that were subjected to mild or strong stress conditions immediately after training.

	Total object exploration time (s)	Number of crossings	Number of rearings
Mild stress			
Vehicle	26.9 ± 4.6	19.2 ± 2.5	45.6 ± 5.7
Amphetamine 0.5	37.2 ± 7.6	20.4 ± 2.7	42.0 ± 4.1
Amphetamine 1	26.5 ± 3.1	17.1 ± 2.4	40.7 ± 5.4
Strong stress			
Vehicle	40.9 ± 5.4	30.5 ± 4.0	51.9 ± 5.0
Amphetamine 0.5	39.8 ± 4.8	22.1 ± 2.2	42.5 ± 4.3
Amphetamine 1	36.1 ± 6.3	23.7 ± 3.0	41.2 ± 5.0
Sham			
Mild Stress			
Vehicle	36.0 ± 5.7	19.7 ± 2.7	46.0 ± 5.9
Amphetamine 1	34.6 ± 4.7	17.1 ± 2.7	36.6 ± 4.3
Strong Stress			
Vehicle	44.4 ± 4.7	14.7 ± 1.8	23.4 ± 1.9
Amphetamine 1	36.9 ± 5.2	14.1 ± 1.9	21.8 ± 2.3
Medullectomy			
Mild Stress			
Vehicle	49.2 ± 10.9	18.0 ± 3.5	58.5 ± 11.5
Amphetamine 1	38.0 ± 4.6	15.3 ± 2.5	43.2 ± 6.6
Strong Stress			
Vehicle	49.9 ± 7.9	15.0 ± 1.9	24.0 ± 3.3
Amphetamine 1	48.5 ± 6.0	19.2 ± 2.3	27.5 ± 3.4

### Amphetamine Impairs Long-Term Memory Consolidation in Adrenal Medullectomized Rats Subject to the Mild Stress Condition

In this experiment we sought to determine whether amphetamine enhancing effects on long-term memory consolidation in rats exposed to the mild forced swim stress condition were dependent on the activation of the peripheral adrenergic system.

Training Trial. Two-way ANOVA for total exploration time of the two identical objects on the training trial revealed a significant adrenal medullectomy effect (F_(1,33)_ = 5.947, *p* = 0.020), but no significant treatment (F_(1,33)_ = 0.136, *p* = 0.715) or the interaction between these two factors (F_(1,33)_ = 1.252, *p* = 0.271) effects (Table 1). Two-way ANOVA for the number of crossings and rearings revealed no significant effects of post-training drug treatment (crossings: F_(1,33)_ = 0.052, *p* = 0.821; rearings: F_(1,33)_ = 0.137, *p* = 0.714), adrenal medullectomy (crossings: F_(1,33)_ = 0.261, *p* = 0.613; rearings: F_(1,33)_ = 0.284, *p* = 0.598) or the interaction between these two factors (crossings: F_(1,33)_ = 0.384, *p* = 0.540; rearings: F_(1,33)_ = 0.058, *p* = 0.812) (Table 1).

**FIGURE 3:**
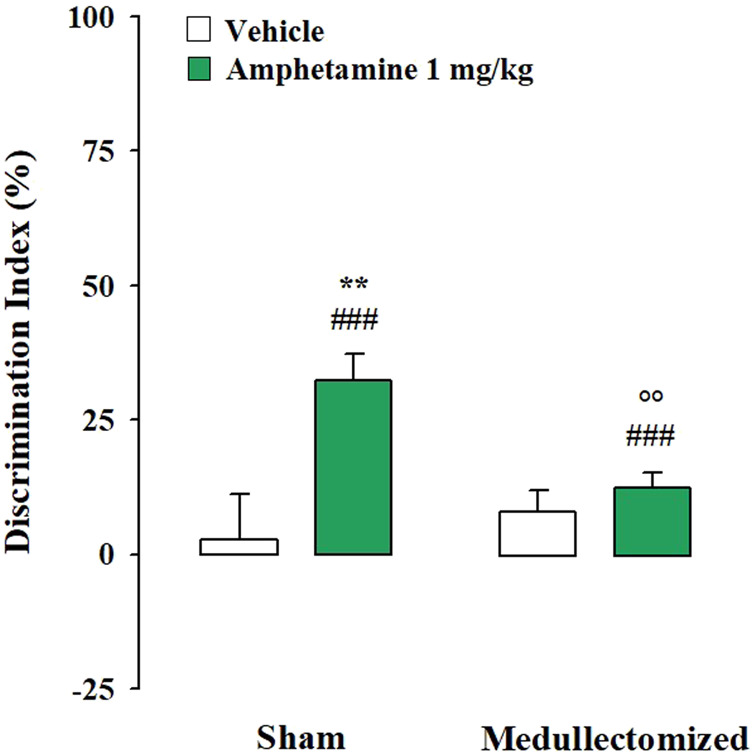
Influence of the peripheral adrenergic system on amphetamine effects on the long-term OR memory consolidation in rats exposed to the mild stress condition immediately after training. DI on the testing trial for sham and medullectomized rats that were treated with vehicle or amphetamine and subjected to the mild stress condition immediately after training. *Post hoc* comparisons reported significant differences between groups as follows: **p < 0.01 vs the corresponding vehicle group. °*p* < 0.01 vs. the corresponding sham group. ###*p* < 0.001, one-sample t-test significantly different from zero. Data are expressed as mean ± SEM (n = 8–13 per group).

Testing Trial. One sample t-test revealed that the DIs of both sham and medullectomized rats that were treated with vehicle were no significantly different from zero (sham: t_(7)_ = 0.774, *p* = 0.464; medullectomized: t_(7)_ = 2.007, *p* = 0.085), thus indicating that both experimental groups were not able to express long-term retention for the familiar object. On the contrary, the DIs of both sham and medullectomized animals treated with amphetamine were significantly different from zero (sham: t_(7)_ = 8.423, *p* < 0.0001; medullectomized: t_(12)_ = 4.519, *p* = 0.0007), thus suggesting that both experimental groups were able to discriminate the two objects. Two-way ANOVA for the DI revealed significant effects for treatment (F_(1,33)_ = 11.329, *p* = 0.002), adrenal medullectomy (F_(1,33)_ = 4.538, *p* = 0.041) and the interaction between both factors (F_(1,33)_ = 6.081, *p* = 0.019). As expected, post hoc analysis revealed that sham rats treated with amphetamine showed higher DI with respect to sham rats treated with vehicle (*p* < 0.01). Surprisingly, post hoc analysis indicated that medullectomized rats treated with amphetamine showed lower DI than the respective sham group (*p* < 0.01) (Figure 3). No significant statistical differences were found between medullectomized rats treated with amphetamine and their respective vehicle treated group. Finally, rats’ exploratory behavior of the apparatus during the testing trial did not differ among the different experimental groups. Indeed, two-way ANOVA did not express any significant effects for total object exploration time (treatment: F_(1,33)_ = 0.941, *p* = 0.339; adrenal medullectomy: F_(1,33)_ = 1.674, *p* = 0.205; treatment × adrenal medullectomy: F_(1,33)_ = 0.582, *p* = 0.451), the number of crossings (treatment: F_(1,33)_ = 0.838, *p* = 0.367; adrenal medullectomy: F_(1,33)_ = 0.377, *p* = 0.543; treatment × adrenal medullectomy: F_(1,33)_ = 0.001, *p* = 0.991) and rearings (treatment: F_(1,33)_ = 2.590, *p* = 0.117; adrenal medullectomy: F_(1,33)_ = 1.557, *p* = 0.221; treatment × adrenal medullectomy: F_(1,33)_ = 0.148, *p* = 0.703) (Table 2).

### Amphetamine Enhanced Long Term Memory Consolidation in Adrenal Medullectomized Rats Subject to the Strong Stress Condition

In this experiment we sought to determine whether amphetamine impairing effects on long-term memory consolidation in rats exposed to the strong forced swim stress condition were dependent on the activation of the adrenergic system.

Training Trial. Two-way ANOVA for total object exploration time on the training trial revealed no significant post-training treatment (F_(1,40)_ = 2.243, *p* = 0.142), adrenal medullectomy (F_(1,40)_ = 0.073, *p* = 0.788), or treatment × adrenal medullectomy (F_(1,40)_ = 0.029, *p* = 0.865) effects. Two-way ANOVA for the number of crossings on the training trial revealed a significant adrenal medullectomy effect (F_(1,40)_ = 8.158, *p* = 0.007), but no significant treatment (F_(1,40)_ = 0.751, *p* = 0.391), or adrenal medullectomy × treatment (F_(1,40)_ = 0.610, *p* = 0.440) effects. Concerning the number of rearings, two-way ANOVA reported no significant effects of treatment (F_(1,40)_ = 0.532, *p* = 0.470), adrenal medullectomy (F_(1,40)_ = 0.507, *p* = 0.481) or interaction between these two factors (F_(1,40)_ = 1.683, *p* = 0.202) (Table 1).

**FIGURE 4:**
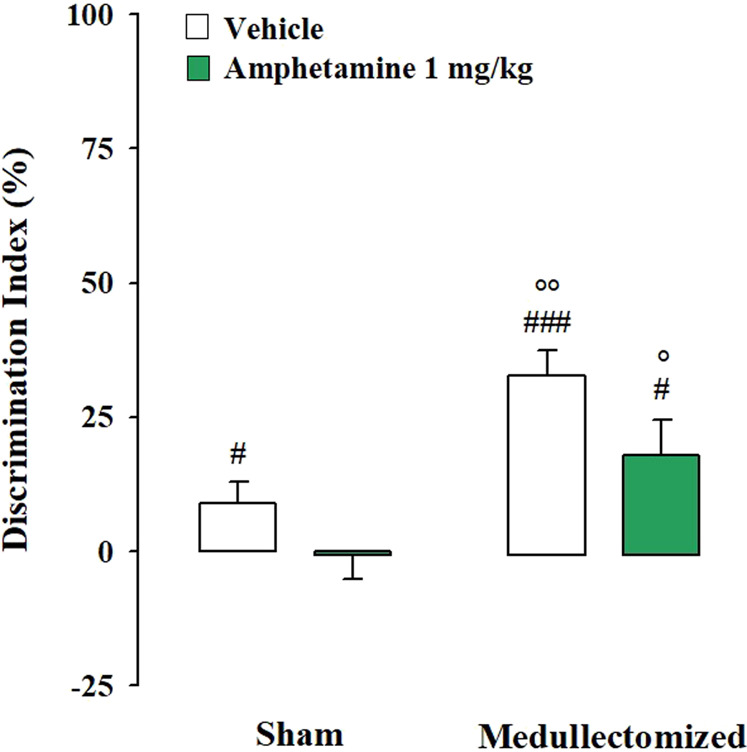
Influence of the peripheral adrenergic system on amphetamine effects on the long-term OR memory consolidation in rats exposed to the strong stress condition immediately after training. DI on the testing trial for sham and medullectomized rats that were treated with vehicle or amphetamine and subjected to the strong stress condition immediately after training. *Post hoc* comparisons reported significant differences between groups as follows: °*p* < 0.05; °*p* < 0.01 vs the corresponding sham group. #*p* < 0.05; ###*p* < 0.001, one-sample t-test significantly different from zero. Data are expressed as mean ± SEM (n = 10–12 per group).

Testing Trial. One sample t-test revealed that in the sham group, only vehicle-treated animals were able to express long-term memory retention for the familiar object (sham: t_(9)_ = 2.275, *p* = 0.049; amphetamine: t_(11)_ = -0.127, *p* = 0.901). In the medullectomized groups, rats treated with vehicle or with amphetamine significantly discriminated the two objects (sham: t_(10)_ = 7.003, *p* < 0.0001; amphetamine: t_(10)_ = 2.775, *p* = 0.020). Two-way ANOVA for the DI revealed significant effects of treatment and adrenal medullectomy (F_(1,40)_ = 5.662, *p* = 0.022; F_(1,40)_ = 17.932, *p* = 0.0001, respectively), but no significant effect for the interaction between both factors (F_(1,40)_ = 0.264, *p* = 0.610). Post hoc analysis indicated that medullectomized animals treated with vehicle or amphetamine showed higher DIs with respect to the corresponding sham groups (*p* < 0.01; *p* < 0.05, respectively) (Figure 4). Conversely, no significant statistical differences were revealed between sham rats treated with amphetamine and their respective vehicle-treated group, as well as among medullectomized groups. Concerning rats’ exploratory behavior of the experimental apparatus during the testing trial, two-way ANOVA indicated no significant effects for total object exploration time (treatment: F_(1,40)_ = 0.453, *p* = 0.505; adrenal medullectomy: F_(1,40)_ = 1.777, *p* = 0.190; treatment × adrenal medullectomy: F_(1,40)_ = 0.319, *p* = 0.575), number of crossings (treatment: F_(1,40)_ = 0.753, *p* = 0.391; adrenal medullectomy: F_(1,40)_ = 1.743, *p* = 0.194; treatment × adrenal medullectomy: F_(1,40)_ = 1.432, *p* = 0.238) and of the number of rearings (treatment: F_(1,40)_ = 0.118, *p* = 0.733; adrenal medullectomy: F_(1,40)_ = 1.232, *p* = 0.274; treatment × adrenal medullectomy: F_(1,40)_ = 0.774, *p* = 0.384) (Table 2).

## Discussion

The present findings show that the psychostimulant amphetamine exerts dichotomic effects on long-term recognition memory, which are strictly dependent on the level of stress experienced soon after encoding. Our results indicate that amphetamine enhances long-term consolidation of recognition memory when rats are exposed to a mild stress condition immediately after training, yet impairing memory performance in case of strong stress exposure.

Over time, amphetamine has become greatly famous for its powerful stimulation properties (Heal et al., 2013). Unfortunately, it has consequently soon became one of the most commonly abused drugs (Berman et al., 2008). The psychostimulant properties of amphetamine depend on its modulation of both the noradrenergic and dopaminergic systems (Fleckenstein et al., 2007). Amphetamine regulation of memory processes has been studied for many decades (Martinez et al., 1983; Oscos et al., 1988; Bardgett et al., 2019). It has been shown that amphetamine-dependent enhancement of memory consolidation depends on the noradrenergic system stimulation properties (Lee and Ma, 1995). Many studies demonstrated that the noradrenergic signaling activation finely regulates cognitive functions (McIntyre et al., 2002; Ferry and McGaugh, 2008; Wichmann et al., 2012), including memory consolidation for emotional experiences (Campolongo et al., 2009a; Roozendaal and McGaugh, 2011; McIntyre et al., 2012; McGaugh, 2013). We very recently demonstrated that post-training administration of amphetamine enhances long-term memory consolidation of an inhibitory avoidance discrimination task, which allows to examine the emotional memory linked to aversive stimuli, and that such effect is totally attributable to the modulation of the noradrenergic tone rather than on any alteration of the dopaminergic system (Colucci et al., 2019). Previous data reported a time-dependent effect of amphetamine on memory storage processes (Packard and McGaugh, 1994; Simon and Setlow, 2006). Particularly, it has been shown that post-training administration of amphetamine selectively enhances memory consolidation in both spatial and cued discrimination water maze tasks without affecting test performance *per se* (Packard and McGaugh, 1994).

During stress response, the HPA axis is activated and different stress mediators and modulators, such as epinephrine, glucocorticoids (i.e., cortisol in humans and corticosterone in rodents) and endocannabinoids are released and act as endogenous modulators of memory consolidation (McIntyre and Roozendaal, 2007; Campolongo et al., 2009b; Campolongo et al., 2012; Morena and Campolongo, 2014; Morena et al., 2014; Atsak et al., 2015; Morena et al., 2015; Morena et al., 2016). It is well known that the relationship between stress exposure and memory function follows an inverted U-shaped curve in which memory performance increases with optimal levels of stress (Salehi et al., 2010). An inverted U-shaped dose-response curve has also been documented for amphetamine effects on memory processes, similarly to several other adrenergic agents (Krivanek and McGaugh, 1969; Baldi and Bucherelli, 2005). Our results that amphetamine influences rat long-term recognition memory consolidation, without any interference of unspecific factors (e.g., sensorimotor, attentional), in a stress intensity-dependent fashion reinforce this evidence and highlight the existence of a modulatory interaction between amphetamine and different stress intensities in the modulation of long-term memory consolidation. This dichotomic effect could be explained in view of the noradrenergic modulation of memory, which is influenced by both amphetamine administration and stress experience and considering the inverted U-shape dose-response curve induced by norepinephrine on memory performance (Baldi and Bucherelli, 2005). Our results demonstrate that exposure to mild stress, immediately after the training trial of an OR task, prevents rats from expressing long-term memory retention for the familiar object. However, this effect is counteracted by post-training administration of amphetamine, which enhances long-term recognition memory retention. Accordingly, previous findings indicated that both amphetamine and stress are able to enhance norepinephrine brain levels (Valentino et al., 1993; Ferrucci et al., 2019). Hence, it is tentative to speculate that the norepinephrine levels elicited by exposure to a mild stress condition are not sufficient to enhance memory consolidation processes, but that treatment with amphetamine, specifically at the higher dose of 1 mg/kg, raises norepinephrine to a critical level able to enhance long-term memory consolidation; further studies aimed at disentangling this issue are therefore demanding.

If a mild stress experience is not *per se* sufficient to create a long-term memory trace of the training trial, a more intensive stress, experienced immediately after training, is able to induce long-term memory retention of the training experience (Santori et al., 2019). Conversely, the concurrent treatment with amphetamine leads to a long-term memory consolidation impairment. Therefore, it can be hypothesized that if the strong stress condition enhances the norepinephrine concentration to a critical level, able to create a long-term trace of the training experience, the treatment with amphetamine, combined with a strong stress experience, induces a norepinephrine release which strongly exceeds the optimal levels, leading to an impairment of long-term memory consolidation.

Previous evidence demonstrated that amphetamine administration completely block the forced swim stress-induced expression of the corticotropin-releasing hormone (hnCRH) and it partially reduce c-fos expression in the paraventricular nucleus of the hypothalamus (PVN), indicating that a negative synergy between amphetamine and stress occurs dampening the characteristic peripheral physiological response to stress and activation of the PVN (Gomez-Roman et al., 2016). However, it has also been shown that amphetamine administration augmented the plasma adrenocorticotropin (ACTH) levels and HPA hormone concentrations, such as epinephrine and glucocorticoids (Gomez-Roman et al., 2016). Early studies suggested a key role of epinephrine in the modulation of norepinephrine release in the brain (Gold and van Buskirk, 1978). Epinephrine is not able to cross the blood-brain barrier and its central effects are due to the stimulation of β-adrenoceptors on vagal afferents terminating in the nucleus of the solitary tract (NTS) (Roozendaal and McGaugh, 2011). NTS innervate the Nucleus Paragigantocellularis (PGi) and other brain regions; PGi sends excitatory fibers, to the Locus Coeruleus (LC); in turn, LC sends noradrenergic projections to many brain areas involved in the modulation of memory consolidation (Roozendaal and McGaugh, 2011).

Previous findings have demonstrated that surgical removal of adrenal medulla abolishes the amphetamine enhancing effects on memory consolidation in rats not exposed to any stressful condition (Martinez et al., 1980b), thus demonstrating that amphetamine effects on memory consolidation are mediated by the peripheral adrenergic tone. In the second set of experiments, we therefore aimed at examining the potential role of the peripheral adrenergic tone in the modulation of long-term memory consolidation exerted by amphetamine administration after different stress intensities experienced soon after learning. Our results clearly indicate that the peripheral adrenergic system plays a key role in the amphetamine modulatory effects on memory. Particularly, here we found that medullectomy not only was sufficient to block the amphetamine enhancing effects on memory consolidation in rats exposed to mild stress condition, but it impaired memory performance; on the contrary, exposure to strong stress alone immediately after training ameliorated long-term memory retention. There is thus tentative to speculate that the stress intensity-dependent epinephrine release alters, through the vagal nerve-NTS-PGi-LC pathway, the norepinephrine transmission in the brain. Such influence, together with the amphetamine-mediated modulation of the noradrenergic system, finely tunes norepinephrine release in specific brain areas crucially involved in memory consolidation (e.g., hippocampus, amygdala), determining, according to the norepinephrine dose-response U-shaped curve, either impairing or enhancing effects on long-term memory consolidation.

Disruption of memory function is seen in a number of stress-associated disorder such as post-traumatic stress disorder (PTSD) (Berardi et al., 2014; Morena et al., 2018; Watson, 2019). Many studies indicated that the noradrenergic system might be responsible for the persistence of traumatic memories in PTSD (Debiec et al., 2011; Gazarini et al., 2014; Liu et al., 2019). A hallmark feature of such psychiatric condition is the over-consolidation of the traumatic experience, which in turn leads to maladaptive behavior (Desmedt et al., 2015). Exaggerated memories are generally potentiated by drug of abuse consumption (Colucci et al., 2019; Gisquet-Verrier and Le Dorze, 2019). Increases of norepinephrine contents were detected in response to both amphetamine administration, known to stimulate the noradrenergic system, and after exposure to trauma and its relative reminders (Le Dorze et al., 2019). Growing evidence supports a crucial link between psychostimulant abuse and PTSD development (Ruglass et al., 2014; Crum-Cianflone et al., 2015). On the other side of the coin, it has to be taken into consideration, that stress often exacerbates neuropsychiatric symptoms of several disorders, such as attention deficit hyperactivity disorder (ADHD) and in turn, neuropsychiatric disorders may cause a continuous state of stress. Memory problems are a frequently reported symptom in adult with ADHD, a disorder often treated with amphetamine or amphetamine-derived drugs. It is well-documented that adults with ADHD perform poorly not only in working memory tasks but also on long-term memory tests (Skodzik et al., 2017). Our results, showing that amphetamine enhances long term memory in animals exposed to mild stressful conditions but impairs it if the level of stress is too high, could be of help to the clinicians to apply a personalized pharmacological therapy in ADHD treatment. Similar considerations could apply when amphetamine is used as a medication in other pathologies strictly linked to stress such as obesity and chronic pain (Dalal and Melzack, 1988; Ricca et al., 2009).

The here presented results highlight that amphetamine induces dichotomic effects on long-term memory consolidation, by activating the peripheral adrenergic system, which in turns finely tunes memory performance according to the level of stress experienced immediately after learning. Our findings pave the road to further investigations of a possible amphetamine contribution to the modulation of the mechanisms underlying stress-related disorders.

## Data Availability

The raw data supporting the conclusions of this article will be made available by the authors, without undue reservation.

## Nomenclature

ACTH: Adrenocorticotropin
DAT: Dopamine re-uptake transporter
DI: Discrimination index
hnCRH: Corticotropin-releasing hormone
HPA: Hypothalamic–pituitary–adrenal axis
LC: Locus coeruleus
NET: Norepinephrine re-uptake transporter
NTS: Nucleus of the solitary tract
OR: Object recognition
PGi: Nucleus paragigantocellularis
PVN: Paraventricular nucleus of the hypothalamus
SEM: Standard error of the mean
SERT: Serotonin re-uptake transporter
